# Editorial: Perspectives in Dealing With Surplus Male Farm Animals

**DOI:** 10.3389/fvets.2021.797081

**Published:** 2021-11-12

**Authors:** Mona F. Giersberg, David Renaud, Nicole Kemper

**Affiliations:** ^1^Animals in Science and Society, Department Population Health Sciences, Faculty of Veterinary Medicine, Utrecht University, Utrecht, Netherlands; ^2^Department of Population Medicine, University of Guelph, Guelph, ON, Canada; ^3^Institute for Animal Hygiene, Animal Welfare and Farm Animal Behaviour, University of Veterinary Medicine Hannover, Hanover, Germany

**Keywords:** day-old chicks, dual-purpose breeds, farm animal welfare, sexed semen, veal calves, buck kids

“Sex matters”—this also holds true for animals kept for farming purposes, where female and male animals of most farm animal species are used and managed in different ways. This applies to breeding animals, but also to individuals of highly specialized hybrids and breeds, which produce food for human consumption directly. The production of milk and eggs for instance relies on female reproductive abilities. However, even in these specialized hybrids and breeds, about half of the offspring will usually be male. Due to an antagonism between reproductive and fattening traits, male animals from these specialized strains are not suitable for commercial meat production. From an economic point of view, culling these “surplus” male animals is often more efficient than rearing them. In recent years, the practice of routinely killing large numbers of young, healthy animals has raised moral concerns in society. Due to the shifting status of animals in society, there is a need to develop alternatives for dealing with surplus male farm animals, a variety of which is presented in the contributions to this Research Topic.

The impression of being seen as mere by-products or waste is most strongly manifested in the standard practice of killing day-old male chicks of layer hybrids. Therefore, this practice raises major socio-ethical concerns and is critically discussed or even banned in some countries (1, 2). De Haas et al. conducted an online survey of the Dutch public to assess the awareness of the routine killing of male layer chicks. Even more important with regard to a transfer into practice are their questions on possible alternatives. Is one alternative favored, and are consumers willing to pay more for poultry products that do not require culling day-old male chicks?

There are currently three alternatives to avoid the killing of male chicks: in-ovo sex determination, rearing layer cockerels, and using dual-purpose chickens. The latter of which is receiving increased attention due to its potential to also alleviate several welfare issues associated with conventional high yielding hybrids. In dual-purpose chickens, animals of both sexes gain economic value: the females are kept for egg-laying purposes, whereas the males are reared for meat. However, little is known about the behavior of these hybrids or breeds in conventional housing environments, which were originally designed for specialized hybrids. Malchow and Schrader contribute to closing this gap by investigating the effects of elevated platforms on several behavioral and welfare indicators in male dual-purpose chickens. In contrast, Rieke et al. focused on the potential benefits of dual-purpose hens: are they as prone to feather pecking behavior as conventional layer hybrids? Staying with the females, Daş et al. provided important knowledge on the impact of nematode infections on the immune responses in previously vaccinated dual-purpose hens. These new insights into dual-purpose chickens' behavior, welfare and health are essential for developing and applying animal-friendly husbandry practices.

The cattle sector faces a similar issue as the poultry industry. Here, specialized breeds are used for dairy or beef production. Male calves of dairy breeds are often only kept for a short period at the lowest costs possible until they are sold or slaughtered, which—in addition to the socio-ethical concerns mentioned above—can lead to serious welfare problems. In their narrative review, Creutzinger et al. detail the welfare challenges associated with raising surplus dairy calves in the U.S. and Canada. The authors highlight that the dairy industry is not only faced with surplus male calves but also with female calves which are not needed as replacements for the lactating herd. In this sense, the case of surplus dairy calves is framed as a “wicked problem” by Bolton and von Keyserlingk. It is demonstrated that such a “wicked problem” calls for approaches that go beyond simple technical solutions. In the methodologies proposed by the authors, integrating the views of the public is key. In this context, Maher et al. surveyed an important yet often underrepresented stakeholder group: the farmers. With the recent expansion of dairy herds in Ireland, farmers also have to deal with an increase in the number of surplus calves. Knowing farmers' views and opinions on how to deal with male dairy calves is a valuable first step for developing alternative approaches that will actually be implemented into practice.

A practical approach to improve the welfare of surplus dairy calves during the short period they are kept is proposed by Boyle and Mee. The authors argue that providing farmers with feedback of results from animal-based welfare indicators collected around slaughter could protect and improve on-farm calf welfare. Therefore, they present a risk-orientated ante- and post-mortem welfare assessment scheme tailored to calves which are younger than 1 month of age. With their investigations of different treatments during transport from the dairy to the veal farm, Marcato et al. join the idea of safeguarding the welfare and health of surplus calves. Are welfare and health affected by the diet the calves are fed prior to transport, by transport duration, or by the type of vehicle used for transport?

Another practical approach to dealing with surplus dairy calves is proposed by Rutherford et al.: as beef consumption increased in the UK over the past decade, why not use male dairy calves to meet this demand in a sustainable way? In their article, the authors give recommendations on which beef production system would fit best the welfare requirements of the dairy calves and the UK market. In contrast, Balzani et al. suggest the use of sexed semen as an option to decrease the number of male calves born. They present a pilot study exploring the perceptions of key stakeholders, such as farmers, veterinarians, and dairy farm advisors, regarding the use of sexed semen as a strategy to reduce the production of surplus male dairy calves.

Finally, this Research Topic focuses on another milk producing species: goats. In some regions of the world, for instance in the Netherlands, goats are almost exclusively bred for milk production. For this purpose, only a small population of the male offspring is needed for reproduction aims. Meijer et al. compare various scenarios of dealing with surplus buck kids in a country, in which there is hardly any market for goat meat. The authors base their recommendations on the sector's experiences and current practices in the Netherlands.

Overall, the body of research included in this Research Topic highlights the variety of alternatives for avoiding the production of surplus male animals or their killing without intentional use. By providing science-based approaches for tackling the issue, welfare friendly, socially accepted, and thus sustainable livestock farming practices can be realized. In addition, the studies on the dual-purpose chickens show that while we are searching for alternatives for the males, we may at the same time encounter ways to improve the welfare of the females.

## Author Contributions

All authors listed have made a substantial, direct and intellectual contribution to the work, and approved it for publication.

## Conflict of Interest

The authors declare that the research was conducted in the absence of any commercial or financial relationships that could be construed as a potential conflict of interest.

## Publisher's Note

All claims expressed in this article are solely those of the authors and do not necessarily represent those of their affiliated organizations, or those of the publisher, the editors and the reviewers. Any product that may be evaluated in this article, or claim that may be made by its manufacturer, is not guaranteed or endorsed by the publisher.
